# Analyzing the Relationship between Solid-Phase Molecular Presentation and Cell Proliferation, Morphology and Secretion Using CellStudio

**DOI:** 10.1021/acsami.5c18270

**Published:** 2025-12-29

**Authors:** Enrique Azuaje-Hualde, Naiara Lartitegui-Meneses, Juncal Alonso-Cabrera, Yara Alvarez-Braña, Marian Martínez de Pancorbo, Fernando Benito-Lopez, Lourdes Basabe-Desmonts

**Affiliations:** † Microfluidics Cluster UPV/EHU, BIOMICs Microfluidics Group, 82992 University of the Basque Country UPV/EHU, Vitoria-Gasteiz 01006, Spain; ‡ Microfluidics Cluster UPV/EHU, Analytical Microsystems & Materials for Lab-on-a-Chip (AMMa-LOAC) Group, University of the Basque Country UPV/EHU, Leioa 48080, Spain; § BIOMICs Research Group, Lascaray Research Center, University of the Basque Country UPV/EHU, Vitoria-Gasteiz 01006, Spain; ∥ Basque Foundation of Science, IKERBASQUE, Bilbao 48080, Spain

**Keywords:** cell microenvironment, solid-phase, growth factor presentation, biosensing, secretomics, mesenchymal stem cells

## Abstract

Precise control over the spatial presentation of molecular cues is essential to engineer functional cell microenvironments. We previously developed CellStudio, a bead-based microsystem that combines cell patterning with localized biosensing, a cell analysis platform for monitoring cell secretion with spatial resolution. In this study, we extend its capabilities to investigate how the solid-phase presentation of fibroblast growth factor 2 influences cell behavior. By customizing the ratio of fibroblast growth factor 2-functionalized microbeads surrounding patterned mesenchymal stem cells clusters, we demonstrate dose-dependent effects on cell proliferation, survival, and morphology. Solid-phase fibroblast growth factor 2 presentation induced cytoskeletal remodeling, increased cell spreading and elongation, and early upregulation of fibroblast growth factor receptor 1 membrane expression. Additionally, we show that local fibroblast growth factor 2 stimulation enhances the secretion of vascular endothelial growth factor, which was *in situ* captured using functionalized bead sensors integrated into the same substrate. This study demonstrates how CellStudio enables parallel, high-resolution analysis of cellular response to growth factor presentation, providing a versatile platform for investigating cell-microenvironment interactions with potential applications in regenerative medicine, drug screening, and basic cell biology.

## Graphical abstract

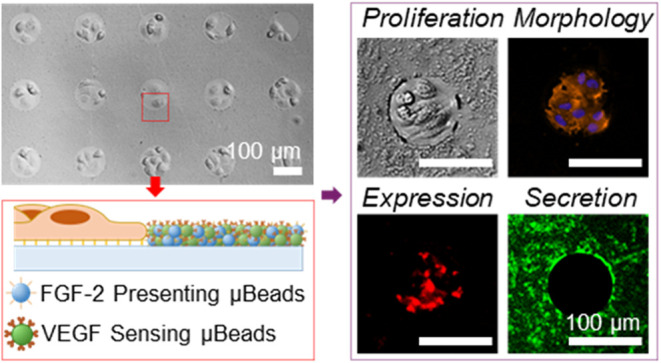

## 1 Introduction

Cells reside in a complex microenvironment composed of both neighboring cells and the surrounding extracellular matrix (ECM).^1,2^ This environment is defined by complex interactions, including direct cell–cell communication through physical contact and the release of signaling molecules such as growth factors, cytokines, and hormones.^3^ These soluble factors play a critical role in regulating cellular behavior, interacting synergistically with both cells and the ECM by mediating signaling through liquid- and solid-phase interactions, which are essential for controlling cellular activities such as growth, differentiation, and migration.^4,5^ Conventional cell culture techniques, the gold standard for in vitro studies, offer limited control over these interactions. In traditional cultures, cells typically grow randomly on flat surfaces, where soluble factors are distributed throughout the liquid medium, failing to replicate the structured and spatially precise solid-phase interactions found in living tissues.^6,7^ Additionally, traditional cell culture methods rely on external assays, such as ELISA, to analyze the secretion of signaling molecules. These assays require removal of the entire supernatant, making it impossible to link molecule secretion to specific interactions within the cell microenvironment.^8^

Recent efforts to develop platforms that better replicate physiological microenvironments have focused on enabling more comprehensive analyses of cell behavior.^9–11^ However, many of these platforms still share key limitations with traditional cell culture methods. In particular, they often lack integrated capabilities to detect and analyze molecular secretion, especially soluble factors.^12,13^ Among the few platforms that integrate secretion sensing, most focus on single-cell analysis,^14–17^ primarily emphasizing cell positioning rather than broader cell–cell interactions, failing to capture the complex interplay between cells and their environment. Recently, a few systems have been developed to replicate controlled physiological conditions while also incorporating cell secretion analysis. Notable examples include the development of a liver-on-a-chip model for electrochemical detection of transforming growth factor,^18^ an adipose tissue model for cytokine detection via surface plasmon resonance (SPR),^19^ and a pancreas-on-a-chip system for insulin detection using Raman spectroscopy and SPR.^20,21^

While these cell analysis platforms represent a significant advancement in analytical capabilities, they also introduce new challenges due to their complexity. Many systems require specialized equipment and expertise, making them less accessible to researchers. In addition, their rigid design limits flexibility and reduces applicability across diverse biological studies. This complexity makes them less compatible with routine laboratory workflows, highlighting the need for user-friendly technologies that offer tighter control over cell interactions and improved analytical capabilities for more accurate analysis of cell behavior.^13^

To address these challenges, we developed CellStudio, a modular and adaptable platform that overcomes the limitations of both, conventional and advanced cell culture systems, by directly integrating biosensing capabilities into the culture environment.^22^ CellStudio substrates consist of hundreds of individual cell clusters surrounded by microbeads (Figure 1A). This configuration enables spatially resolved monitoring of secreted molecules while allowing precise control over fundamental cell interactions. By combining established techniques such as Printing and Vacuum Lithography (PnVlitho)^23^ with bead-based assays,^24,25^ CellStudio provides a practical solution for studying cells under highly controlled conditions. A key advantage of CellStudio is its modular design, which allows customization for diverse experimental needs. It supports hundreds of individual cell clusters anchored to protein dots that serve as precise adhesion points, enabling control over cell adhesion, distribution, and cell–cell contact.^26–28^ Surrounding each dot, a tridimensional pattern of microbeads is generated. These microbeads can be functionalized with bioreceptors, enabling the direct capture and analysis of cell-secreted molecules without the need for external assays, making easier to study the relationship between cellular processes and molecular signaling.

**Figure 1:**
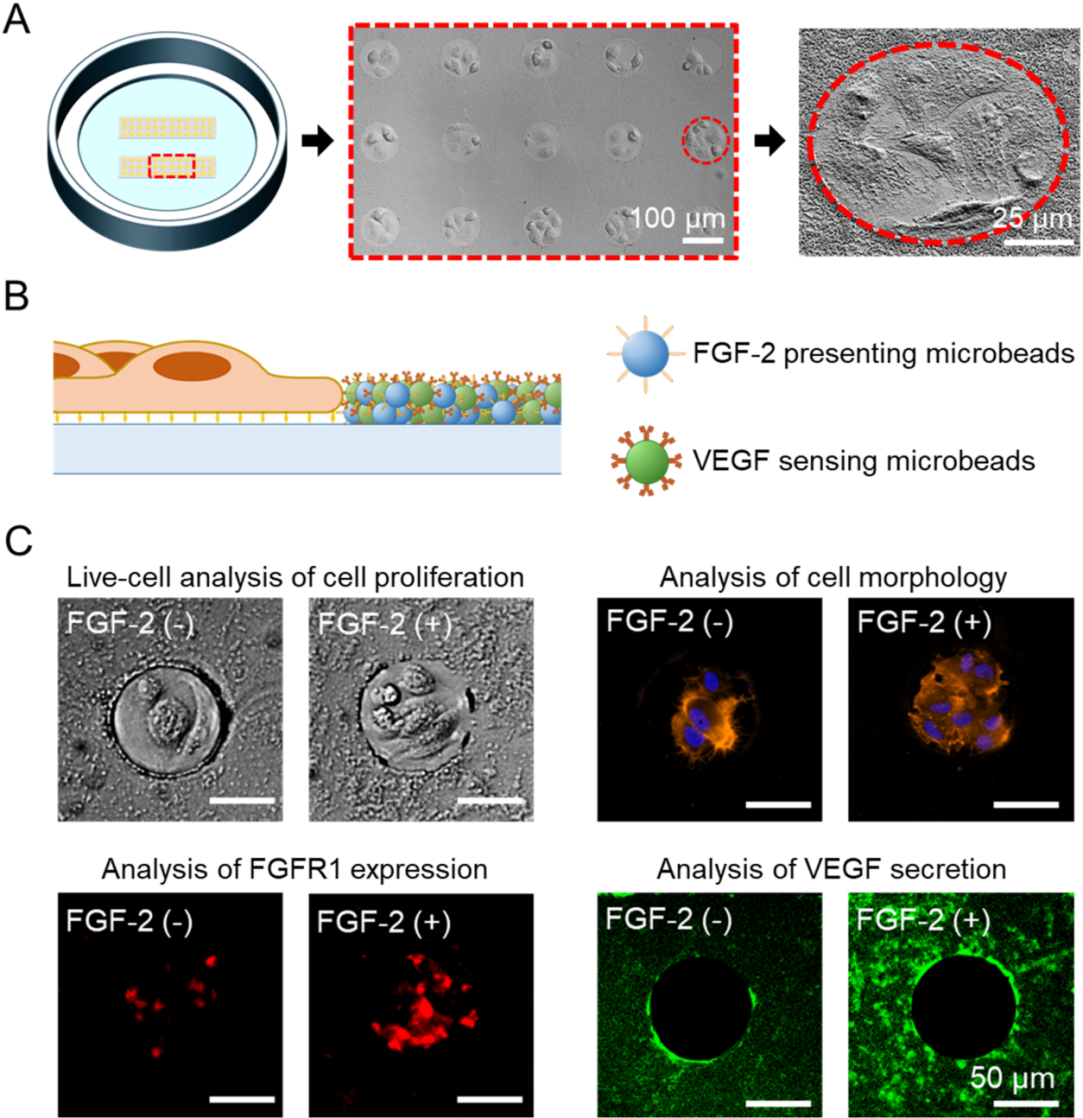
Analysis of cells behavior upon solid-phase presentation of FGF-2 in CellStudio substrates. (A) Drawing, brightfield and SEM images of a CellStudio substrate, an array showing 15 organized cell clusters surrounded by microbeads, and a single cell cluster. (B) Drawing of a cell cluster surrounded by a mixture of FGF-2 functionalized microbeads, acting as presenting beads, and anti-VEGF IgG functionalized microbeads, acting as sensing beads. (C) Brightfield and fluorescence images of the analysis of MSCs behavior upon FGF-2 solid presentation on CellStudio, including live-cell analysis of cell proliferation, and fluorescence staining-based analysis of cell morphology, FGFR1 membrane expression and VEGF secretion.

In this work, we extend the capabilities of CellStudio by also using microbeads as carriers for molecules of interest, such as growth factors, allowing solid-phase presentation directly around individual cell clusters. Because each microbead functions as an independent presentation or sensing unit, mixtures of differently functionalized beads can be positioned around the cells, providing an intuitive and tunable way to control the local dose of a stimulus while simultaneously measuring the resulting cellular response. Additionally, this approach enables the integration of microbeads with both stimulating and sensing capabilities within the same pattern, Figure 1B, creating a unique type of analytical assay in which the secretion of one factor can be analyzed in response to the presentation of another.

To validate the technology, we tested the capabilities of the CellStudio platform by using Fibroblast Growth Factor 2 (FGF-2) as a model growth factor, described to anchor the ECM in *in vivo* conditions.^29,30^ FGF-2 is known for its role in promoting the proliferation of mesenchymal stem cells (MSCs) and inducing the expression of specific membrane receptors, such as Fibroblast Growth Factor Receptor 1 (FGFR1).^31–34^ We conducted several analyses within the CellStudio platform to evaluate the effects of solid-phase presentation of FGF-2, immobilized on microbeads, on MSC patterns, demonstrating the diverse range of analytical assays enabled by the platform. These analyses included optical observation of cell proliferation, the fluorescence-based analysis of cell morphology and FGFR1 expression inside the cells, and the novel fluorescence-based analysis of Vascular Endothelial Growth Factor (VEGF) secretion near the cell clusters, Figure 1C.

## 2 Materials and Methods

### 2.1 Preparation of Functionalized Microbeads

Streptavidin coated polystyrene microbeads (0.2 or 0.5 μm diameter, Immunostep, Spain) were employed in all cases. For microbeads functionalization, stock microbead suspensions (volumes between 10–25 μL) were centrifuged at 14,000 rpm/18,800*g* for 9 min on an Eppendorf centrifuge 5425 (Germany). Afterward, the microbeads were resuspended with 60 μL of the corresponding functionalizing solution and kept on constant mixing for 1 h. Functionalizing solutions included: distilled water (DI), for the generation of B_beads_, 5 μg mL^–1^ biotinylated Fibroblast Growth Factor 2 solution (Deltaclon, Spain) in DI, for the generation of FGF-2_beads_, and 5 μg mL^–1^ biotin Polyclonal Goat anti-Vascular Endothelial Growth Factor IgG solution (R&D Systems) in DI, for the generation of anti-VEGF-_beads_. Then, the suspensions were centrifuged at 14,000 rpm for 9 min. The functionalized microbeads were resuspended in distilled water in the necessary volume to obtain a desired final concentration of 6 × 10^11^ microbeads mL^–1^ or 6 × 10^10^ microbeads mL^–1^ for the 0.2 and 0.5 μm diameter microbeads, respectively.

### 2.2 Fabrication of CellStudio Substrates: Co-Patterning of Microbeads and Cell Adhesion Isles

To generate combined patterns of microbeads and cell-adhesion sites, the Printing and Vacuum Lithography (PnVlitho) technique was used. This method integrates microcontact printing with vacuum-driven lithography to spatially organize fibronectin adhesion dots and microbeads on a glass substrate (see Figure 2 and Supporting Information 1).

**Figure 2:**
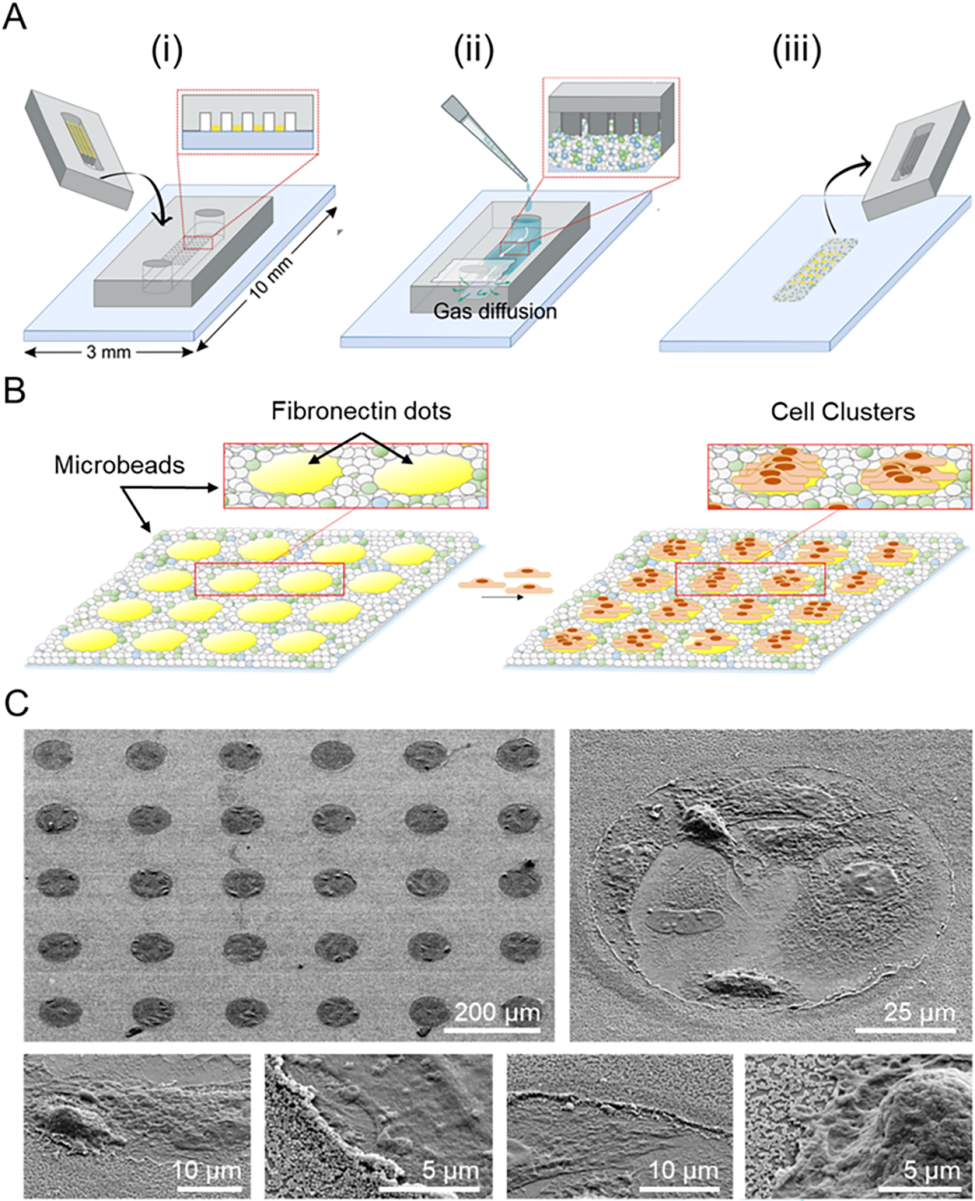
Fabrication of CellStudio substrates trough PnVlitho. (A) Schematic drawing of microcontact printing: pillars within the PDMS stamps are incubated with a cell adhesion protein solution, and after drying, is directly put into contact with the flat glass surface (i). Then, PDMS stamps sealed with the glass substrate are degassed upon vacuum that, when put into normal atmospheric pressure, enables the flow of a microbead suspension between its features (ii). Upon solvent evaporation, the microbeads remain bound to the flat surface (iii). (B) Schematic drawing of cell capture and adhesion onto the fibronectin dots: after PDMS stamp retrieval, CellStudio patterns are incubated with a cell suspension, which results in a multicomponent pattern of small cell clusters surrounded by the microbeads. (C) SEM images of cell loaded CellStudio substrates with increased zoom.

Polydimethylsiloxane (PDMS) slabs containing channel-like structures (1000 μm × 5000 μm × 13 μm, width × length × height) and uniformly spaced pillars (100 μm diameter, 100 μm center-to-center spacing) were employed. These pillars served both as microcontact printing features and as obstacles that define the bead flow paths. In all cases, the PDMS channels were punched to generate a 2 mm inlet and a 1 mm outlet. For microcontact printing, the pillars were incubated with a 50 μg mL^–1^ fibronectin solution for 30 min to define the cell-adhesive regions. After incubation, the PDMS slabs were rinsed with distilled water, dried with compressed air, and placed in conformal contact with the plasma-oxidized glass bottoms of custom PMMA wells. Dry transfer of fibronectin from the pillars produced an array of protein dots on the glass surface; in this study, the configuration generated arrays of 250 fibronectin dots per well.

Vacuum-driven lithography was then used to position microbeads around the printed protein dots. The PDMS–glass assemblies were placed in a desiccator at 0.7 mbar for 20 min to degas the PDMS. After vacuum release, the outlet was temporarily sealed, and 2 μL of the functionalized microbead suspension was added to the inlet. As air diffused back into the PDMS, a controlled negative pressure drew the suspension through the channel and between the pillars, guiding the beads into the spaces surrounding the protein dots. The outlet acted as a reservoir where the carrier fluid accumulated first, preventing the beads and fluid from separating during flow and ensuring that the bead suspension entered the patterned area uniformly. The suspension was allowed to propagate until it reached the outlet. After 5 min, the tape at the outlet was removed and the assembly was left overnight at 4 °C to allow solvent evaporation. After evaporation, the PDMS slab was removed, leaving fibronectin dots surrounded by a dense microbead layer on the glass surface (30 ± 2 and 5 ± 1 microbeads μm^2^, for 0.2 and 0.5 μm diameter microbeads, respectively). The microbead pattern could consist of either a single bead type or a homogeneous mixture of beads with different functionalizations, depending on the experiment. Finally, each well was filled with 1 mL of blocking solution (5% BSA, 10% casein, 0.2% milk powder in PBS) and incubated for 2 h before cell seeding.

### 2.3 Cell Seeding and Cell Culture on CellStudio Substrates

For mesenchymal stem cells patterning, hair Human Follicle Mesenchymal Stem Cells (MSCs, isolated from one human donor follicles) were detached from the flasks and were resuspended in serum free Dulbecco’s Modified Eagle’s medium (serum-free medium (SFM), Fisher Scientific, Spain) at a concentration of 10^5^ cells mL^–1^. Then, the blocking solution was removed and the PMMA wells were rinsed three times with PBS. 750 μL of the cell suspension was loaded into them. To ensure specific cell adhesion to the fibronectin dots, the wells were left inside the incubator (37 °C, 5% CO_2_) on constant oscillation using a rocker (Vari-Mix steep angle rocker, Thermo Fisher) for 120 min. Afterward, the medium was retrieved and the wells were rinsed three times with PBS to wash out any nonattached cell. For cell maintenance throughout all experiments, the cell-loaded CellStudio substrates were incubated with 750 μL of SFM.

### 2.4 Immunostaining of FGFR1

After 5 h incubation of the MSCs clusters in the incubator (37 °C, 5% CO_2_), cells were fixed with paraformaldehyde 4% (Panreac Quimica, Spain) for 10 min. Samples were incubated with a BSA solution 1% for 1 h, and subsequently incubated with a solution of primary anti-FGFR1 rabbit antibody 5 μg mL^–1^ (Fisher Scientific, Spain), BSA 0.2% (w/v) and Goat Serum 1% (v/v, Fisher Scientific, Spain) in PBS for 2 h. Then, the samples were rinsed with PBS three times and incubated with a secondary antibody solution containing Goat antirabbit IgG Alexa Fluor 647 5 μg mL^–1^ (Fisher Scientific, Spain), BSA 0.2% (w/v) and Goat Serum 1% (v/v) for 45 min. Finally, the samples were rinsed three times with PBS and analyzed.

### 2.5 Immunostaining of Secreted VEGF

For VEGF, after 48 h in the incubator (37 °C, 5% CO_2_), MSCs cells on the patterned substrates were fixed by incubation with paraformaldehyde 4% (Panreac Quimica, Spain) for 10 min at room temperature. Then cells were dyed with phalloidin Alexa Fluor 594 (Fisher Scientific, Spain) for 30 min and with DAPI (Fisher Scientific, Spain) for 5 min, following manufacturers protocol. Samples were then incubated with 400 μL of a 1 μg mL^–1^ Mouse Monoclonal anti-VEGF IgG (R&D Systems) with 10% Goat Serum (v/v), 0.2% milk powder (w/v) in PBS for 45 min. Then, the samples were rinsed with PBS three times and incubated with a secondary antibody solution containing 1 μg mL^–1^ Goat anti-Mouse IgG Alexa Fluor 488 (Fisher Scientific, Spain) for another 20 min. Finally, the samples were rinsed three times with PBS and analyzed.

### 2.6 Image and Data Analysis

For the characterization of microbeads patterns, CellStudio substrates were imaged through Scanning Electron Microscopy (SEM) using a HitachiS-4800 SEM microscope.

The number of cells in each cell cluster were manually quantified from brightfield microscopy images. For the analysis of the fluorescence intensity in the vicinity of each spot, an annular region of interest (ROI) was used. This ROI had an inner diameter of 100 μm and an outer diameter 110 μm, covering an area 10 μm wide from the edge of the protein spot. Brightfield and fluorescence microscope images were taken with a modified Nikon Eclipse TE2000-S inverted microscope (USA), with and adapted Andor Zyla sCMOS black and white camera (Oxford Instruments, UK) and lumencor laser for excitation and Quad EM filter: 446/523/600/677 with 4 TM bands: 446/34, 523/42, 600/36, 677/28. Further fluorescence microscope images were taken with an Olympus IXplore SpinSR10 spinning disk adapted to a camera HAMAMATSU ORCA FusionBT and widefield filters Ex: BP 352-402 | Em: LP 409, Ex: BP 460-480 | Em: BP 495-540, Ex: BP 530-550 | Em: LP 575 and Ex: BP 565-585 | Em: BP 600-690.

Microscopy images were processed by FiJi/ImageJ software.^35^ Data and Statistical analysis were performed in Excel 2016 and GraphPad Prism 8.

## 3 Results and Discussion

### 3.1 Fabrication of CellStudio Using Printing and Vacuum Lithography

The CellStudio substrates are fabricated using a technique known as PnVlitho, described in Section 2.2., following a similar protocol than the one described by Azuaje-Hualde et al.^22^ This process combines microcontact printing and vacuum-driven lithography to fabricate precise, customizable cell culture substrates, allowing controlled cell adhesion and spatial organization of microbeads that serve as both sensors and molecular carriers, see Figure 2A.

CellStudio substrates consist on a two-dimensional pattern of protein dots remains on the substrate, encircled by a three-dimensional arrangement of microbeads. This configuration enables precise spatial control of cell adhesion, resulting in an array of uniformly spaced cell clusters encircled by microbeads (Figure 2B), enabling direct interaction between the cell clusters and the surrounding beads. More detailed information can be found in Supporting Information 1. The microbead pattern can consist of a homogeneous mixture of microbeads with different functionalizations (see Supporting Information 2). Figure 2C presents SEM images of a CellStudio substrate section at different magnifications after incubation with MSCs, illustrating the spatial arrangement of cell clusters, the homogeneous distribution of microbeads on the surface, and the direct physical contact between cells and beads.

### 3.2 Analysis of MSC’s Proliferation and Survival upon Solid-Phase Presentation of FGF-2 in CellStudio Substrates

FGF-2 is a key growth factor that stimulates MSC growth and proliferation.^34,36–38^ Within the CellStudio platform, the effect of solid-phase FGF-2 presentation at different dosages can be evaluated through simple live-cell microscopy. The experimental setup consisted of MSC clusters patterned on an array of 100 μm fibronectin dots, surrounded by microbeads (500 nm ø) functionalized with FGF-2. Three microbead patterns were designed to assess the effect of different FGF-2 dosages on MSC behavior (Figure 3A). The first configuration consisted solely of nonfunctionalized microbeads (Blank_beads_), representing the absence of the growth factor (0% FGF-2 substrates). The second included a 1:1 mix of Blank_beads_ and FGF-2-functionalized microbeads (FGF-2_beads_), where half of the microbeads contained the growth factor (50% FGF-2 substrates). The third configuration exclusively used FGF-2_beads_, with all microbeads containing the growth factor (100% FGF-2 substrates). After 24 h of incubation in SFM, the patterns were imaged using brightfield microscopy.

**Figure 3:**
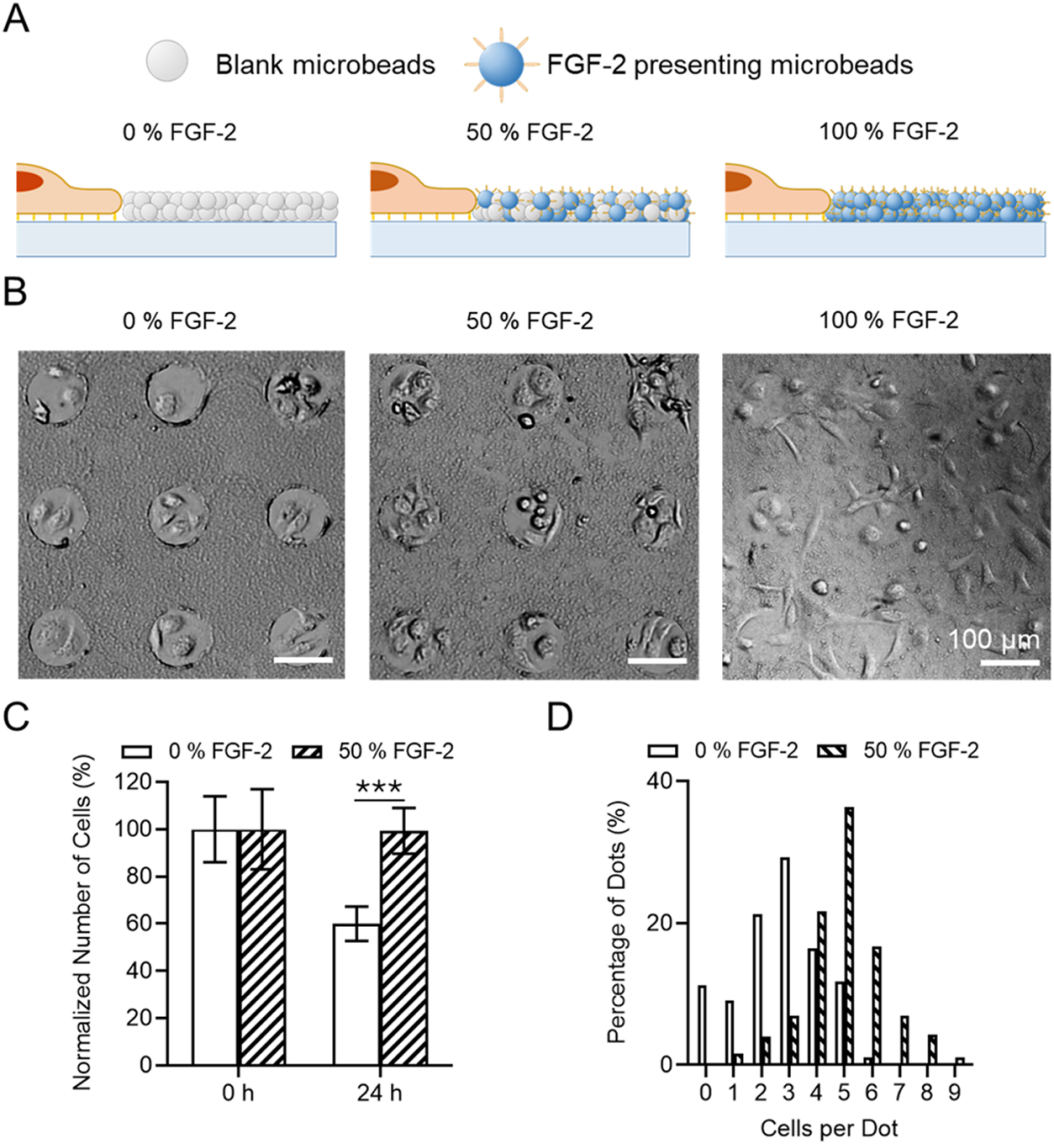
Analysis of MSC’s proliferation and survival upon solid-phase presentation of FGF-2 in CellStudio substrates. (A) Drawing of cell cluster on 100 μm fibronectin dots surrounded by the microbeads pattern containing, from left to right, increasing percentage of FGF-2_beads_ (0, 50, and 100% FGF-2). (B) Brightfield microscope images of CellStudio substrates containing MSCs cell clusters on 0% FGF-2, 50% FGF-2 and 100% FGF-2 patterns after 24 h incubation in culture media. (C) Plot of the normalized number of cells per array in 0 and 50% FGF-2 patterns after 24 h incubation in culture media. Data for each condition was normalized to the respective number of cells at time 0 h. Error bars indicate mean value ± SEM (*n* = 200 cell clusters from 3 CellStudio substrates). Statistical significance; nonparametric Mann–Whitney test (***p < 0.001). (D) Plot of the percentage of the number of cells in each the dot of the array in 0 and 50% FGF-2 patterns after 24 h incubation in SFM. *Y*-axis indicates, out of the total number of analyzed dots (*n* = 200, defined as 100%), the percentage of dots containing a given number of cells.

MSCs clusters exhibited distinct responses, depending on the concentration of FGF-2_beads_. As shown in Figure 3B, the presence of FGF-2 was associated with a significant increase in cell number within the patterns and distinct changes in cell morphology. A more detailed analysis of individual cell clusters revealed an 80% increase in the number of cells per array in 50% FGF-2 substrates compared to 0% FGF-2 substrates, where cell detachment and death were prevalent due to serum-free conditions (Figure 3C). Most cell clusters in the 50% FGF-2 substrates (75%) contained 4–6 cells per dot, exhibiting an elongated morphology. In contrast, clusters in the 0% FGF-2 substrates mostly (66%) contained 2–4 cells per dot, displaying a more rounded morphology (Figure 3D). The 100% FGF-2 substrates exhibited overstimulation, leading to outward growth and migration of cell clusters beyond their original confined dot. The expansion and migration of cells were only observed under solid-phase FGF-2 exposure and are consistent with the increased spreading and motility reported for MSCs under strong FGF-2 stimulation. In association with this migration, local reductions in bead density occasionally appeared near migrating cells in the 50 and 100% FGF-2 substrates. These findings reinforce the role of FGF-2_beads_ in enhancing MSC proliferation and survival, highlighting the dosage-dependent effects of FGF-2 on MSC behavior, demonstrating the capability of CellStudio to precisely control exposure through microbead composition.

Results obtained with the CellStudio platform followed the expected trend of FGF-2–driven stimulation that we first verified in conventional cell culture assays (Supporting Information 3). In these plate-based experiments, supplying FGF-2 either in solution (10 ng mL^–1^) or coated onto the surface led to 29 and 60% increases in cell number, respectively, confirming that our MSC line responds to the growth factor as reported in the literature. These conventional cultures serve as a reference confirming that the responses observed in CellStudio are in line with the known effects of FGF-2 on MSCs. Further work should focus on discerning precisely the individual contributions of dose-dependent solid-phase presentation and confinement within the adhesion dots to the observed effects on cell proliferation.

### 3.3 Analysis of MSC’s Morphology upon Solid-Phase Presentation of FGF-2 in CellStudio Substrates

As previously observed, FGF-2 solid phase presentation induced noticeable changes in MSC morphology. To further investigate this, MSC clusters on CellStudio *0*a*nd 50*% *FGF-2 substrates* were incubated for 24 h in SFM, then fixed and stained with DAPI for nucleus visualization and phalloidin for cytoskeleton observation. Samples were then imaged using fluorescence microscopy.

As expected, fluorescence imaging revealed distinct MSC morphology changes (Figure 4A). Total cell area increased by 60% in MSCs cultured on 50% *FGF-2 substrates* compared to 0% *FGF-2 substrates*, from 1150 ± 50 μm^2^ to 1880 ± 90 μm^2^ (Figure 4B). Significant differences in aspect ratio (length/width) were observed as well, with MSCs on 50% *FGF-2 substrates* exhibiting a higher ratio (2.8 ± 0.2) than those on 0% *FGF-2 substrates* (2.0 ± 0.1), indicating enhanced elongation in response to FGF-2 (Figure 4C). The observed increase in cell size and elongation suggests that localized, substrate-bound FGF-2 presentation through the microbeads triggers cytoskeletal remodeling and altered cell spreading behavior.

**Figure 4:**
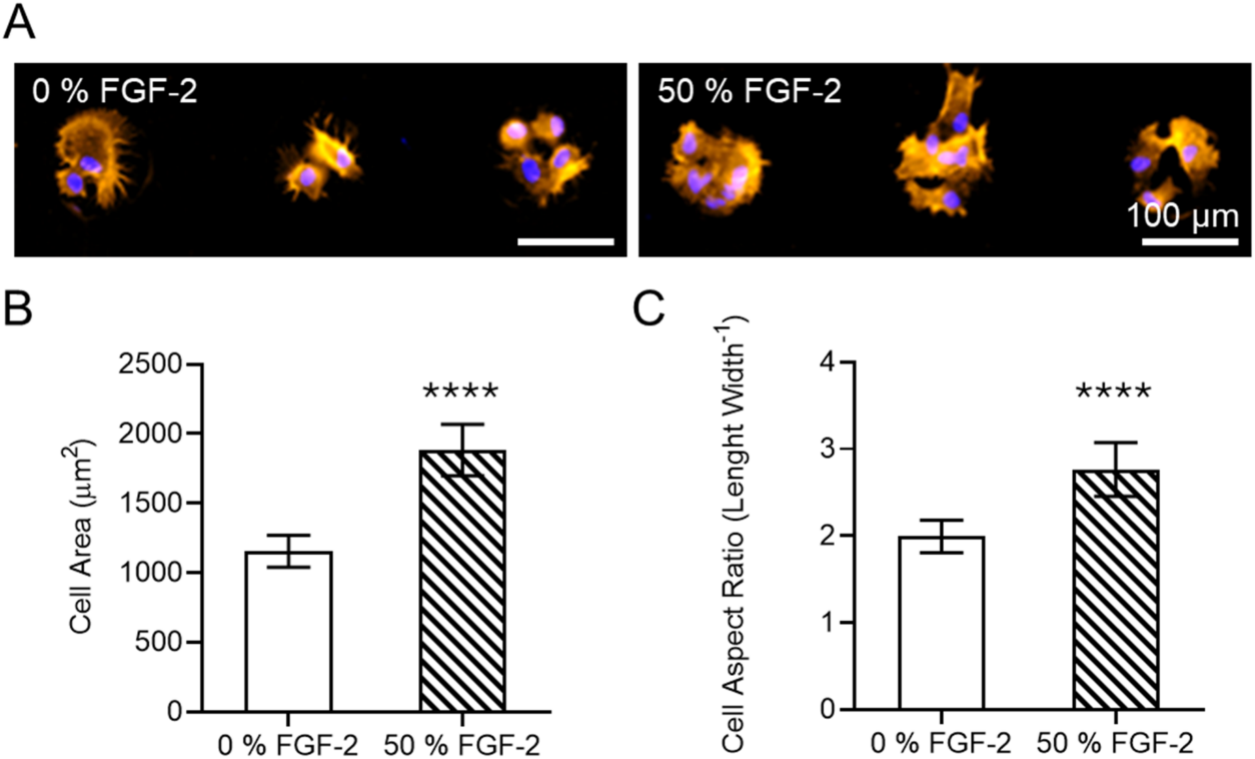
Analysis of MSC’s morphology upon solid-phase presentation of FGF-2 in CellStudio substrates. (A) Fluorescence microscopy images of MSCs clusters on 0 and 50% FGF-2 patterns after 24 h incubation in culture media, showing cytoskeleton staining (Phalloidin, orange), nucleus staining (DAPI, blue). (B) Plot of the mean cell area for 0 and 50% FGF-2 patterns (*n* = 100 cells from 3 patterns). (C) Plot of the mean cell aspect ratio (length divided by width) for 0 and 50% FGF-2 patterns (*n* = 100 cells from 3 CellStudio substrates).

### 3.4 Analysis of Membrane Expression of FGFR1 on MSCs Clusters upon Solid-Phase Presentation of FGF-2 in CellStudio Substrates

FGFR1 is a membrane receptor that can be upregulated in MSCs within hours of FGF-2 exposure.^34^ To expand the analytical capabilities of CellStudio, FGFR1 was labeled in both *0* *and 50*% *FGF-2 substrates* (500 nm ø microbeads) to investigate how the growth factor influences receptor expression. MSCs clusters were cultured for 5 h in SFM, fixed, labeled with an anti-FGFR1 antibody, and imaged using fluorescence microscopy.

Notably, as early as 5 h of incubation, FGFR1 expression significantly increased in response to FGF-2 presentation (Figure 5A). Fluorescence intensity within clusters in the 50% *FGF-2 substrates* was twice that of the 0% *FGF-2 substrates*, confirming the stimulatory effect of FGF-2 on receptor production by individual cells (Figure 5B). This increase was not observed in conventional cell cultures, where FGF-2 was provided in soluble form and did not produce detectable changes in FGFR1 fluorescence compared to untreated controls. These results further validate the effect of FGF-2 presentation on MSCs behavior and highlight CellStudio’s capability to monitor and analyze intracellular events through fluorescence labeling integrated with the solid-phase presentation assay.

**Figure 5:**
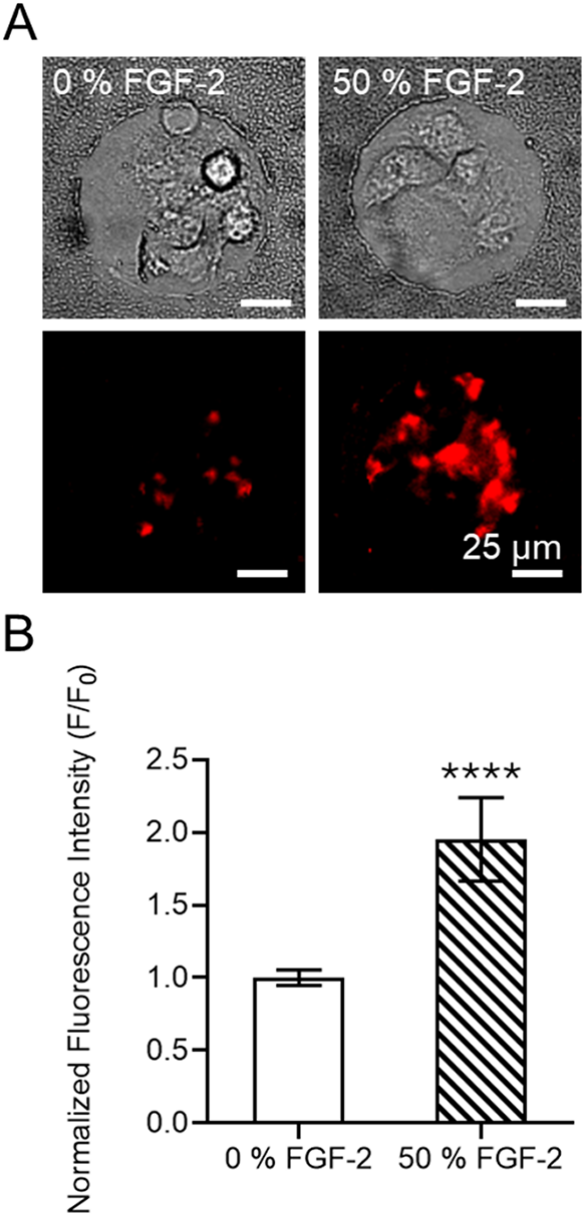
Analysis of membrane expression of FGFR-1 on MSCs clusters upon solid-phase presentation of FGF-2 in CellStudio substrates. (A) Brightfield and fluorescence microscopy images of MSCs clusters on 0 and 50% FGF-2 patterns after 5 h incubation in culture media, stained with anti FGFR-1 Alexa Fluor 647 antibody. (B) Plot of the normalized fluorescence intensity of the cell clusters in 0 and 50% FGF-2 patterns. Fluorescence values were normalized to F_0_, corresponding to the mean fluorescence intensity of 0% FGF-2. Each point represents a single cell cluster (*n* = 25 cell cluster from 3 CellStudio substrates). Statistical significance; nonparametric Mann–Whitney test (****p < 0.0001).

### 3.5 Analysis of VEGF Secretion from MSCs Clusters upon Solid-Phase Presentation of FGF-2 in CellStudio Substrates

As previously demonstrated, CellStudio enables the exposure of cells to direct stimuli using microbeads functionalized with specific signaling molecules. Additionally, microbeads can be functionalized with bioreceptors, such as antibodies, to directly capture, detect, and analyze cell secretion in proximity to each cell cluster. Previously, we employed CellStudio substrates for high-throughput, spatially resolved analysis of cell secretion and diffusion, specifically validating the technology for assessing VEGF secretion from MSCs.^22^ VEGF is a growth factor that induces angiogenesis and vasculogenesis, playing a pivotal role in wound healing and tissue remodeling.^39–41^ Together, FGF-2 and VEGF are key microenvironmental signals that regulate cell adhesion, growth, and proliferation, making them promising therapeutic targets for conditions such as chronic inflammation and cancer.^36,37,42^ However, the regulatory interplay between their expression patterns remains poorly understood.^43,44^

In this context, integrating stimulation and sensing approaches provide a unique means to study cell behavior in vitro, particularly how solid-phase presentation of one factor influences the secretion of another. The modular nature of CellStudio enables the study of cellular responses by combining beads acting as carriers of stimulators or as carriers of bioreceptors, offering a versatile platform for multifaceted experimental investigations. We investigated the integration of solid-phase FGF-2 presentation with direct VEGF secretion detection near patterned cell clusters, enabling the study of how solid-phase FGF-2 presentation modulates VEGF secretion.

Using precise MSCs patterning on 100 μm fibronectin dots, we established two distinct microbead (200 nm ø) configurations (Figure 6A). The first configuration consisted of a 1:1 mix of Blank_beads_ and anti-VEGF IgG-functionalized microbeads (anti-VEGF_beads_), representing the absence of the growth factor (0% *FGF-2 substrates*). The second configuration consisted of a 1:1 mix of FGF-2_beads_ and anti-VEGF_beads_ (50% *FGF-2 substrates*). CellStudio substrates were incubated for 48 h in SFM, immunostained, and imaged via brightfield and fluorescence microscopy to assess cell proliferation and VEGF secretion.

**Figure 6:**
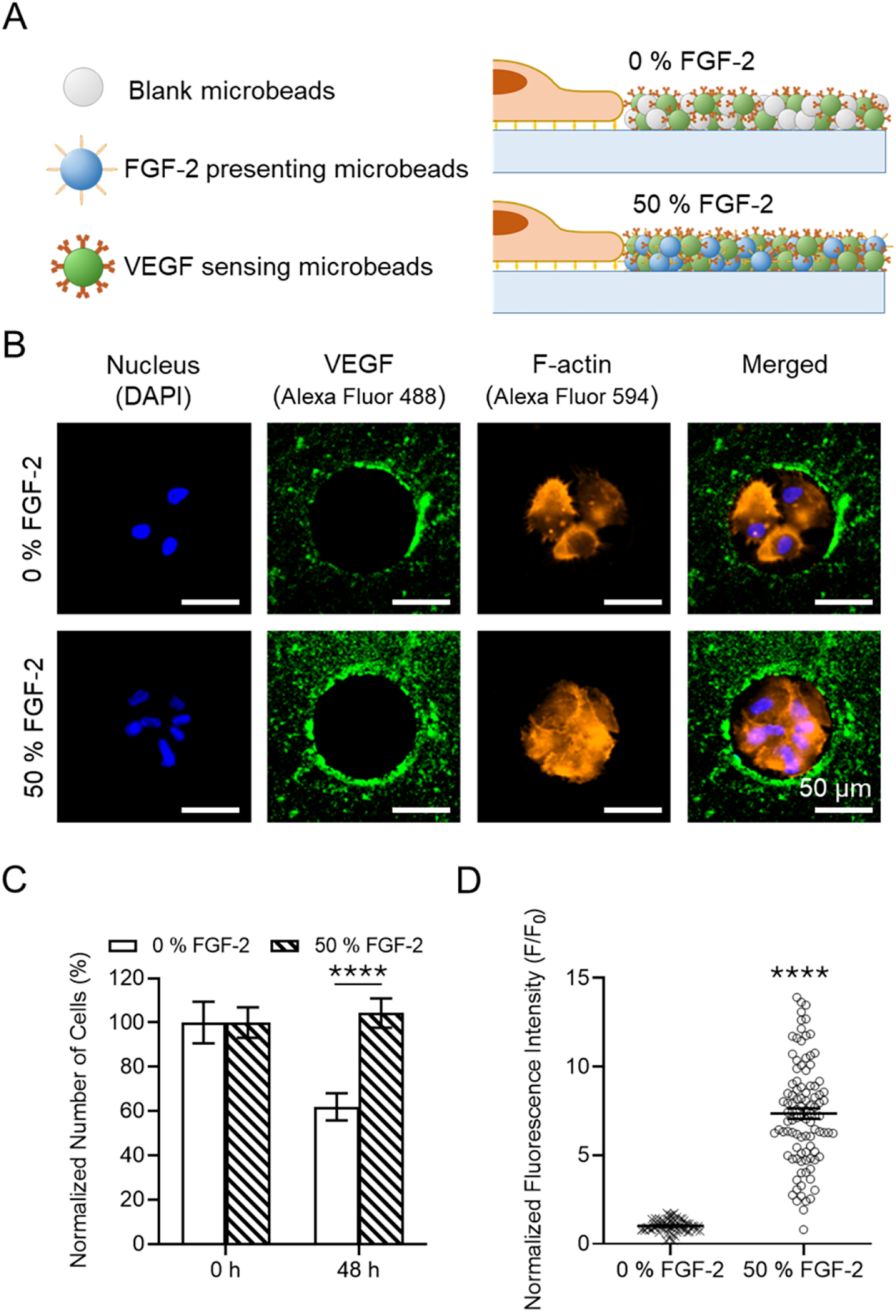
Analysis of VEGF secretion from MSCs clusters upon solid-phase presentation of FGF-2 in CellStudio substrates. (A) Drawing of MSCs clusters surrounded by the microbeads containing anti-VEGF_beads_ and either B_beads_ (0% FGF-2) or FGF-2_beads_ (50% FGF-2). (B) Fluorescence microscopy images of a MSC clusters on 0 and 50% FGF-2 CellStudio substrates, showing cytoskeleton staining (phalloidin, orange), nucleus staining (DAPI, blue) and secreted VEGF staining (Alexa Fluor 488 secondary antibody, green). (C) Plots of the normalized number of cells per array in 0 and 50% FGF-2 patterns after cell seeding and after 48h incubation in culture media. Data for each condition was normalized to the respective number of cells at time 0 h. Error bars indicate mean value ± SEM (*n* = 300 cell clusters from 4 CellStudio substrates). Statistical significance; nonparametric Mann–Whitney test (***p < 0.001). (D) Plot of the fluorescence intensity of the region of interest, see experimental section for explanation, around each cell cluster (10 μm wide ring from the edge of cell clusters, *n* = 100 random cell cluster from 4 CellStudio substrates). Fluorescence values were normalized to F_0_, corresponding to the mean fluorescence intensity of 0% FGF-2 without cells. Statistical significance; nonparametric Mann–Whitney test (****p < 0.0001).

Consistent with our previous assays, our results demonstrated a pronounced cellular response to FGF-2-functionalized microbeads (Figure 6B). This included increased cell proliferation and survival, with 50% *FGF-2 substrates* exhibiting an 80% higher cell count at the end of the assay compared to 0% *FGF-2 substrates*, reaffirming the stimulatory effect of FGF-2 exposure on individual cell clusters (Figure 6C). Moreover, the analysis of VEGF secretion revealed a strong correlation between FGF-2 presence and enhanced VEGF release in the cluster surroundings. The mean fluorescence intensity surrounding FGF-2-exposed cell clusters was 735 ± 30% higher than in untreated MSCs, clearly demonstrating the direct influence of FGF-2 on VEGF secretion regulation (Figure 6D). While, as expected, the presence of FGF-2-functionalized microbeads results in a higher number of VEGF-secreting cells, the pronounced increase in VEGF secretion suggests that FGF-2 stimulation may also upregulate VEGF production at the individual cell level. CellStudio provides a controlled platform to investigate the insufficiently understood interplay between FGF-2 and VEGF, enabling the spatial resolution needed to dissect how growth factor presentation influences signaling. This highlights the platform’s potential for uncovering complex cellular interactions that are difficult to observe using conventional methods.

## 4 Conclusions

CellStudio is a versatile platform for spatially resolved analysis of cell secretion across hundreds of individual clusters. Surface patterning enables precise control over cell–cell interactions, and the microbeads positioned around each cluster allow efficient capture and quantification of secreted molecules such as VEGF. In this study, we expanded CellStudio by incorporating microbeads functionalized with growth factors, enabling solid-phase presentation in close proximity to patterned cells. By mixing different bead types, the local amount of presented growth factor can be adjusted while sensing beads operate in parallel, establishing a simple “present-and-measure” framework. This approach improves control over the cellular microenvironment and provides a practical way to study how localized stimuli influence cell behavior, leading to more accurate experimental outcomes.

Using a model of FGF-2 stimulation in MSCs, we demonstrated that controlled, solid-phase presentation of FGF-2 can be achieved for hundreds of individual clusters within CellStudio, allowing multifaceted analysis of cell behavior. This approach proved to be more effective than conventional *in vitro* assays by improving interactions between cells and growth factors. Additionally, we demonstrated how CellStudio enables the analysis of how one growth factor’s interaction influences the secretion of another, offering a level of sensitivity and control not previously achieved in conventional cell secretion analysis technologies.

In addition to enabling precise control over cell interactions, CellStudio stands out for its simple fabrication and compatibility with standard cell culture and microscopy techniques. This ease of use lowers technical barriers, making it suitable for routine use in biology laboratories. Its effectiveness and adaptability were validated with established models. Future work should extend its application to other cell types, such as cancer models, and enhance its functionality through improvements in pattern design, surface chemistry, and bead diversity.

## Supplementary Material



## Notes

Protocol for the Printing and Vacuum Lithography technique and the fabrication of CellStudio substrates (SI-1); an evaluation of the distribution of microbeads on patterns containing a mix of beads (SI-2); and the experimental results on the stimulation of MSCs with FGF-2 in solution or attached to microbeads in the bottom of a cell culture well (SI-3) (PDF)
