# Supplementary material for: Analyzing the Relationship between Solid-Phase Molecular Presentation and Cell Proliferation, Morphology and Secretion Using CellStudio
Source: ACS Appl Mater Interfaces. 2025 Dec 29;18(4):6423–32. doi: 10.1021/acsami.5c18270 (PMC12884458; doi:10.1021/acsami.5c18270)
Supplement: Supplementary file 1 [file am5c18270_si_001.pdf]

Supporting Information (SI)

Analyzing the Relationship Between Solid-Phase Molecular Presentation and Cell Proliferation, Morphology and Secretion Using CellStudio

Enrique Azuaje-Hualde[1], Naiara Lartitegui-Meneses[1], Juncal Alonso-Cabrera[1,2], Yara Alvarez-Braña[1,2], Marian Martínez de Pancorbo[3], Fernando Benito-Lopez[2]* and Lourdes Basabe-Desmonts[1,2,4]*

Email: Lourdes.basabe@ehu.eus

[1] Microfluidics Cluster UPV/EHU, BIOMICs Microfluidics Group, University of the Basque Country UPV/EHU, Vitoria-Gasteiz, Spain.

[2] Microfluidics Cluster UPV/EHU, Analytical Microsystems & Materials for Lab-on-a-Chip (AMMa-LOAC) Group, University of the Basque Country UPV/EHU, Vitoria-Gasteiz, Spain.

[3] BIOMICs Research Group, Lascaray Research Center, University of the Basque Country UPV/EHU, Vitoria-Gasteiz, Spain

[4] Basque Foundation of Science, IKERBASQUE, Bilbao, Spain

SI-1. Printing and Vacuum Lithography.

CellStudio is based on the development of multicomponent patterns of cells and microbeads, facilitated by a fabrication process called Printing and Vacuum lithography (PnVlitho), **Figure SI-1**. Through the integration of microcontact printing and vacuum-driven lithography, CellStudio enables to achieve precise and reproducible formation of complex patterns on a flat surface (Alonso-Cabrera et al., 2025; Azuaje-Hualde et al., 2025; Hamon et al., 2016).

The initial step in the PnVlitho process involves microcontact printing, which entails the dry-transfer of proteins from a microstructured stamp, made of polydimethylsiloxane (PDMS), onto a surface like glass. In the specific CellStudio configuration utilized in this study, PDMS stamps with uniformly spaced pillars were employed to form an array of fibronectin dots printed onto the substrate, replicating the diameter of the pillars. This step lays the foundation for the precise localization of cell adhesion points within the substrate. In the subsequent stage of the process, the vacuum-driven lithography step, utilizes the gas diffusion properties of PDMS to orchestrate a precisely controlled flow of a microbead suspension within the inter-pillar space of the PDMS stamp. By subjecting the PDMS to a vacuum environment, the air from the polymer is removed. Upon reestablishing regular atmospheric pressure, the PDMS stamp initiates the suction of air from all accessible spaces, including the space in between the carved features. Since the carved side of the PDMS stamp is sealed with the substrate, a controlled flow of a solution or suspension can be generated, facilitated by the resulting negative pressure between the features. In our CellStudio configuration, a suspension of microbeads is introduced, which encircles each PDMS pillar, contributing to the formation of a microbead pattern around the imprinted cell adhesion proteins.

Upon solvent evaporation and retrieval of the PDMS stamp, a two-dimensional pattern of cell adhesion proteins surrounded by a three-dimensional arrangement of microbeads is produced. For our CellStudio configuration, the resulting pattern is comprised of hundreds of fibronectin dots on the surface of a glass substrate homogenously surrounded by the microbeads. When incubated with a cell suspension, cells adhere specifically to the fibronectin dots, generating individual cell clusters, each one being encircled by the surrounding microbeads.

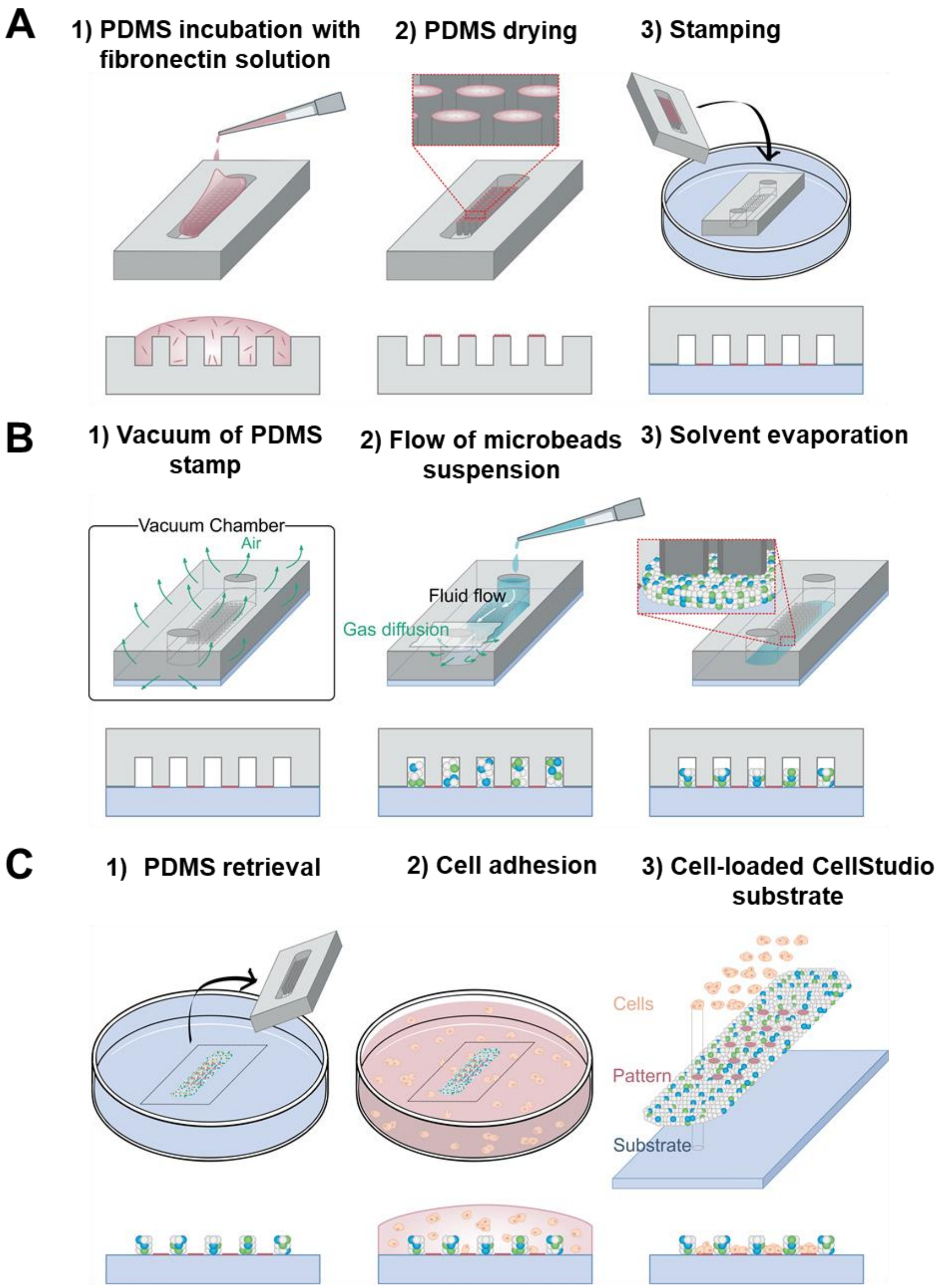

Figure SI-1. Fabrication of CellStudio substrates trough PnVlitho. *A) Schematic drawing of microcontact printing: pillars within the PDMS stamp are incubated with a cell adhesion protein solution (1), and after drying (2), stamp is directly put into contact with the flat surface (3). B)*

Schematic drawing of vacuum-driven lithography of microbeads: PDMS stamps are degassed upon vacuum (1) that when put into normal atmospheric pressure enables the flow of a microbead suspension between its features (2). Upon solvent evaporation, the microbeads remain bound to the flat surface (3). C) Schematic drawing of cell capture and adhesion onto the fibronectin dots: after PDMS stamp retrieval (1), CellStudio patterns are incubated with a cell suspension (2), which results in a multicomponent pattern of small cell clusters surrounded by the microbeads (3).

SI-2. Evaluation of patterns homogeneity when using microbeads mixtures.

In order to obtain information about the dispersion of the microbeads, with different functionalization, within the patterns, microbeads were functionalized with fluorescent biotin as a reporter (R_{beads}). Three different functionalized microbead mixes were prepared: one containing exclusively unfunctionalized beads (B_{beads}, *B_{beads} patterns*), another one containing exclusively R_{beads} (*R_{beads} patterns*) and finally another one consisting of a 1:1 mixture of B_{beads} and R_{beads} (*B_{beads}:R_{beads} patterns*). The different microbeads suspensions were patterned as previously described.

In all cases, the microbeads pattern presented a homogenous distribution (**Figure SI-2**). The fluorescence intensity of the images increased as the concentration of R_{beads} in the sample increased, indicating that an even distribution of the B_{beads} and R_{beads} was achieved. Microbeads patterns did not present differences in terms of aggrupation or agglomerations. This indicated that mixtures of differently functionalized microbeads are properly patterned, enabling the generation of homogeneous layers of microbeads with different functionalizations. In view of these results, it can be presumed that cells attached in cell adhesion zones will have an even contact with each sub-type of microbeads used in the pattern.

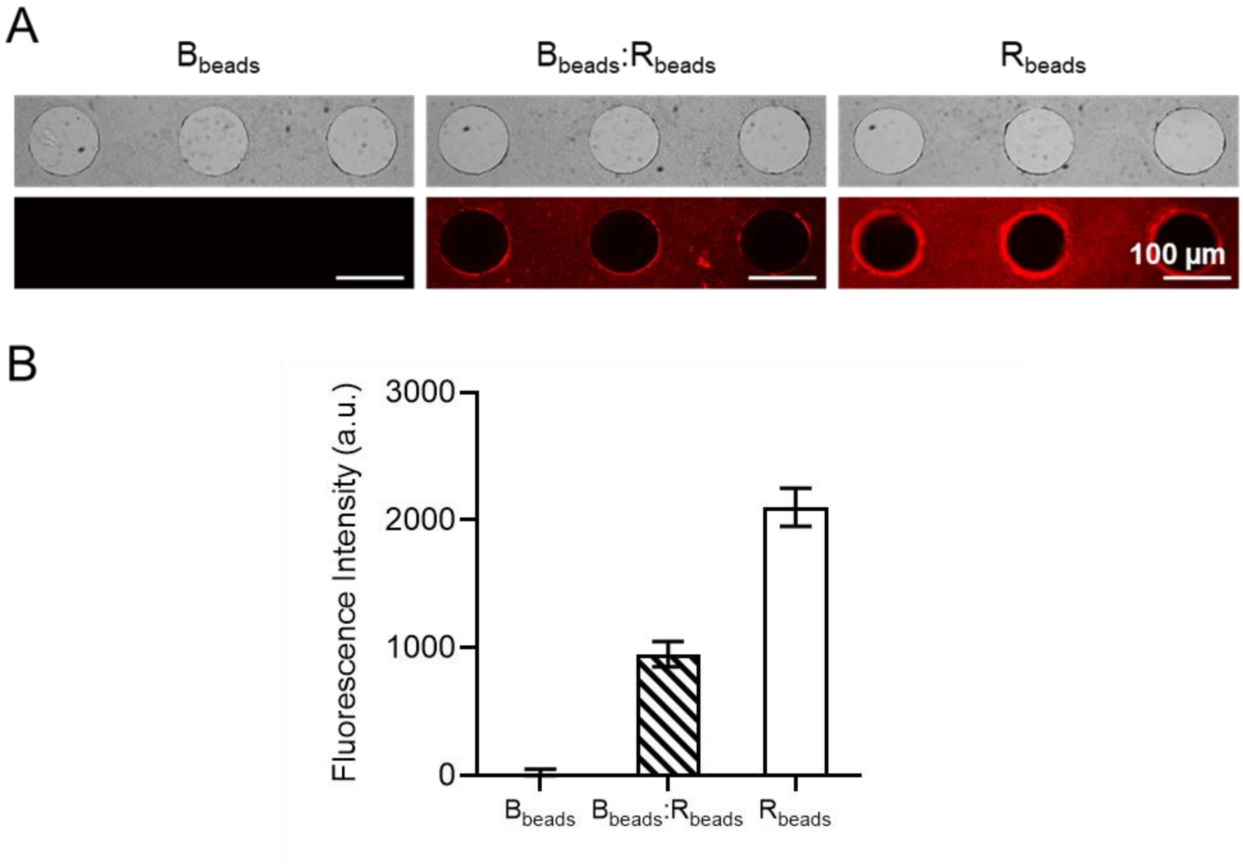

***Figure SI-2. Distribution microbeads patterns containing a mix of beads**. A) Brightfield and fluorescence microscopy images of the patterns containing different percentages of B_{beads} and R_{beads}, fluorescent biotin labelled streptaviding functionalized microbeads. B) Graphical representation of the normalized fluorescence intensity obtained for patterns containing different percentage of fluorescent R_{beads}. (n = 50 dots from 3 patterns)*

SI-3. Fibroblast growth factor 2 (FGF-2) stimulation of mesenchymal stem cells (MSCs) measured by standard protocols.

MSCs seeded on conventional cell culture wells were exposed to FGF-2 either by diluting the growth factor in the medium, or by immobilizing FGF-2 on the bottom of the wells plates prior to cell seeding. For FGF-2 functionalization of the well-plates, 3 wells of a 24-well plate were incubated with 500 µL of streptavidin solution (200 µg mL^{-1}) for 1 h. Afterwards, wells were rinsed with PBS and then incubated with biotinylated FGF-2 (10 ng mL^{-1}) for an additional hour. The rest of the 24 wells remained untreated.

The MSCs (p4) were cultured with Complete Medium (CM) in t75 flaks. The cells were maintained until reaching 80 % confluence for all experiments. 500 µL of the cell suspension of 10^5 cells mL^{-1} in serum-free medium (SFM) were loaded in both treated and non-treated wells (n = 3). Additionally, another three non-treated wells were loaded with 500 µL of the cell suspension of 10^5 cells mL^{-1} in SFM + FGF-2 (10 ng mL^{-1}). Cells were left in the incubator for 24 h. At the end of the assay, cells were photographed and counted.

In all cases, the exposure to FGF-2 resulted in an increased number of cells in the wells at the end of the experiment (**Figure SI-3**). Up to 31 % more cells in the wells treated with 10 ng mL^{-1} FGF-2 dissolved in the suspension ($FGF\text{-}2_{solution}$) than those left untreated (2877 ± 69 and 3606 ± 421 cells per well for untreated cells and cells in $FGF\text{-}2_{solution}$, respectively). Comparing the effect of $FGF\text{-}2_{solution}$ diluted *versus* immobilized FGF-2 on the well plate ($FGF\text{-}2_{fixed}$), up to 29 % more cells could be observed after 24 h (4911 ± 258 cells per well).

Our results validate both that our MSC cell line responds to the presence of FGF-2 as expected, and that solid phase presentation is more efficient than solution-based stimulation, based on the

differences in cell proliferation and morphology found in each case. It should be noted that, cell in the FGF-2_{fixed} wells presented a growth configuration in the form of patches, possibly due to the uncontrolled functionalization of the well plate.

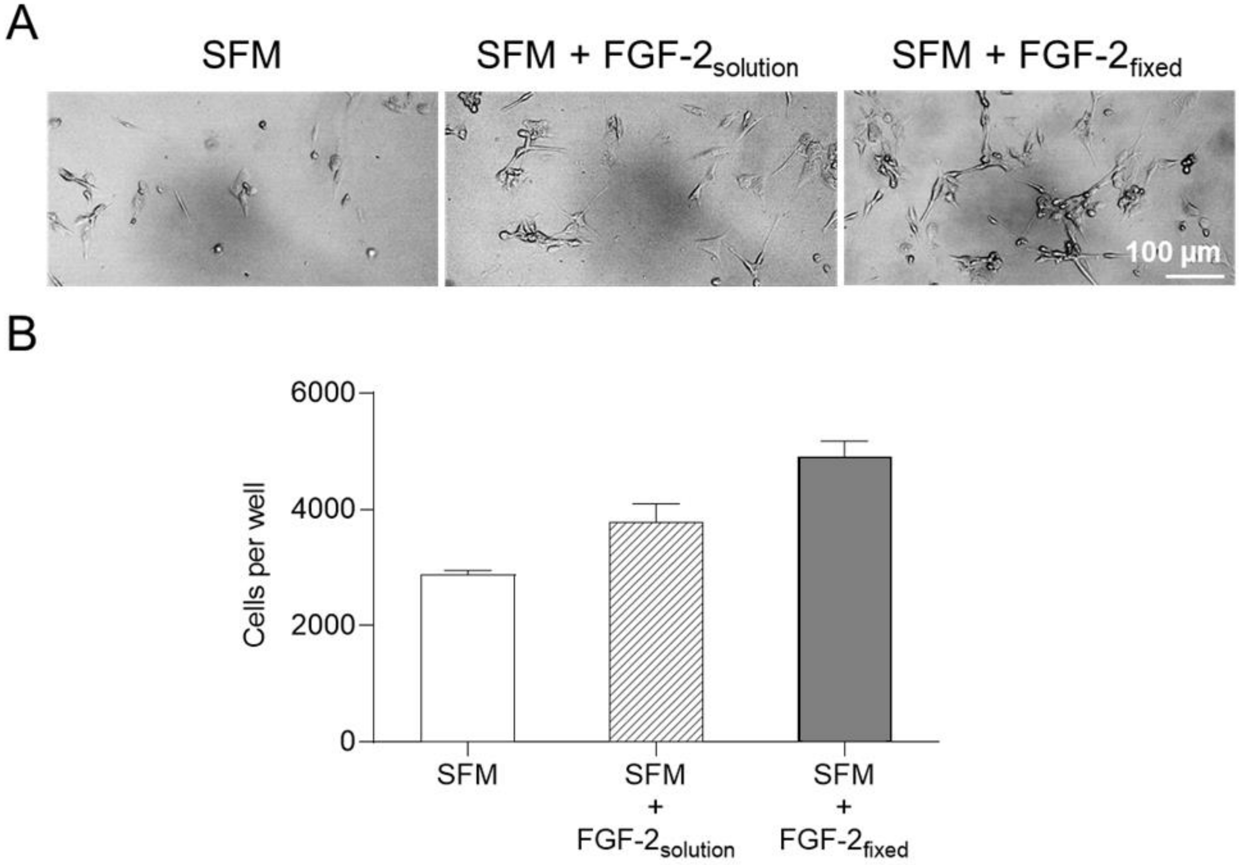

***Figure SI-3. Stimulation of MSCs FGF-2 presentation**. A) Brightfield microscope images of attached MSCs in the different conditions: SFM, SFM + FGF-2 10 ng mL^{-1} in solution (FGF-$2_{solution}$) and SFM + FGF-2 immobilized in the bottom of the well (FGF-2_{fixed}). B) Plot of the number of cells per well when incubated for 24 h in non-treated wells with SFM, FGF-$2_{solution}$ or in FGF-2_{fixed} to the bottom of the wells. Error bars indicate mean valued ± SD (n = 3).*
